# The Construct Structures of Psychological and Behavioral Responses to COVID-19 Pandemic in Pregnant Women

**DOI:** 10.3389/fpsyt.2022.796567

**Published:** 2022-07-12

**Authors:** Zonglin He, Joyce Wai-Ting Chiu, Yuchen Lin, Babatunde Akinwunmi, Tak Hap Wong, Casper J. P. Zhang, Wai-Kit Ming

**Affiliations:** ^1^Department of Infectious Diseases and Public Health, Jockey Club College of Veterinary Medicine and Life Science, City University of Hong Kong, Hong Kong, Hong Kong SAR, China; ^2^Division of Life Science, The Hong Kong University of Science and Technology, Hong Kong, Hong Kong SAR, China; ^3^International School, Jinan University, Guangzhou, China; ^4^Faculty of Biology, Medicine and Health, University of Manchester, Manchester, United Kingdom; ^5^Department of Psychology, University of York, York, United Kingdom; ^6^Department of Psychiatry, The Third Affiliated Hospital of Sun Yat-sen University, Guangzhou, China; ^7^Department of Obstetrics and Gynecology, Brigham and Women's Hospital and Harvard Medical School, Boston, MA, United States; ^8^Center for Genomic Medicine (CGM), Massachusetts General Hospital and Harvard Medical School, Harvard University, Boston, MA, United States; ^9^Department of Obstetrics and Gynecology, The First Affiliated Hospital of Jinan University, Guangzhou, China; ^10^Department of Obstetrics, Guangdong Women and Children Hospital, Guangzhou, China; ^11^School of Public Health, LKS Faculty of Medicine, The University of Hong Kong, Hong Kong, Hong Kong SAR, China

**Keywords:** COVID-19, pregnancy, perinatal depression, post-traumatic stress disorder, structural equating modeling

## Abstract

**Aim:**

The present study aimed to investigate the construct structure behind the psychosocial response, behavioral response, prenatal depression, and post-traumatic stress disorder (PTSD) in pregnant women during the COVID-19 pandemic in China.

**Method:**

The validated Chinese version of the Edinburgh Postnatal Depression Scale (EPDS), PTSD CheckList (PCL)-6, and two newly established scales for COVID-19-related psychological and behavioral responses were used. Structural equation modeling (SEM) analysis was applied to evaluate the structural relationships of psychological and behavioral responses during the COVID-19 pandemic.

**Results:**

Of the 1,908 mothers who completed the questionnaires, 1,099 met the criteria for perinatal depression, and 287 were positively screened for PTSD, where 264 women exceed the cut-off points for both. Pregnant women with full-time or part-time jobs tended to have the lowest scores of EPDS (10.07 ± 5.11, *P* < 0.001) and stress levels (23.85 ± 7.96, *P* = 0.004), yet they were more likely to change their behavior in accordance with the COVID-19 outbreak (13.35 ± 3.42, *P* = 0.025). The structural model fit the data (χ^2^ = 43.260, *p* < 0.001) and resulted in satisfactory fit indices (CFI = 0.984, TLI = 0.959, RMSEA = 0.072, and χ^2^/d*f* = 10.815), all path loadings were significant (*p* < 0.05). The SEM indicates that the level of QoL was attributable to the occurrence of PND, leading to PTSD, and COVID-19 related behavioral and psychological responses.

**Conclusion:**

The inter-relationships between the COVID-19-related psychosocial and behavioral responses have been assessed, indicating that the pandemic increased the burden of perinatal depression. Psychoeducation, as well as other psychological interventions, may be needed to alleviate the COVID-19-based anxiety and increase their engagement in protective behaviors.

## Introduction

A pneumonia-like respiratory system disease outbreak termed the coronavirus disease 2019 (COVID-19) caused by the newly identified coronavirus, the severe acute respiratory syndrome (SARS)-CoV-2, swept through China in early 2020. COVID-19 was officially announced as a global pandemic on 11^th^ March (1). The COVID-19 pandemic has disrupted the lives of millions across the globe, resulting in more than 2 million deaths since January 2020 (2).

The outbreak of infectious diseases induces widespread anxiety (3). During the SARS outbreak in 2003, empirical studies on psychological responses of the general population to epidemics were reported (4–6). Panic over the COVID-19 outbreak resulted in the public over-purchasing and hoarding of necessities, including tissue paper, surgical masks, hand sanitizers, and disinfectants, though such infections do not typically result in death (7, 8). Beyond this, the levels of depression, anxiety, and post-traumatic stress disorder (PTSD) among the public have elevated, pertaining to the infectious disease and the uncertainty of the situation.

Nevertheless, reports of the psychological impact of infectious disease outbreaks on pregnant women have been rare. Pregnant women are particularly affected, as they are naturally concerned about the safety of their fetus and tend to overestimate the risk of contracting the disease (5, 9). Elevated stress level in pregnant women during the SARS outbreak was precipitated by the infection and the epidemic itself, quarantine, economic fallout, and lack of social and family support (3). Previous trauma and recent stressful life events were associated with anxiety and depression in perinatal period (3, 10), yet it impacts pregnancy and postpartum stages differently (11). Psychometric evidence of the Perceived Stress Scale has shown that pregnant women are more associated with depression with certain risk factors, namely unemployment, lower education level, perceived stress in the last month, and recent life adversity (12). Nevertheless, symptoms of both depression and PTSD are prevalent amongst post-delivery mothers (13, 14).

Our preliminary nationwide cross-sectional study on the psychological impact of COVID-19 on pregnant women has revealed a high prevalence of probable depression in prenatal depression (PND) (34%) and suspected PTSD (40%) (15). Although many studies have investigated the relationship between peripartum depression and PTSD in pregnant women, the construct relationship is still not fully elucidated, let alone the interactions during the COVID-19 pandemic (16, 17).

The COVID-19 situation is likely to generate additional stress for perinatal women (18–20). Current studies on COVID-19 have documented anxiety in pregnant women regarding themselves or their friends and family being infected during and after their pregnancy (15). Concerns about miscarriage and preterm births have also been reported, leading these women to consider altering their perinatal care routine, including postponing or canceling perinatal appointments. The implementation of physical distancing and quarantine guidelines might have further disrupted the modalities and delivery of routine prenatal care and impeded access to social support after childbirth (21–23).

Furthermore, the experiences of emotional insecurity may be acute (15, 24), where women might experience intense loneliness from the absence of companionship during the delivery or postpartum care. Hence, sufficient caregiving and other social support are much needed by both their mothers and family.

Coping skills have always been a precipitate and perpetuity for psychiatric disorders (25–27). Empirical evidence has indicated that suppressive, proactive, and avoidant coping strategies, such as behavioral disengagement and problem denial, are more prevalent among patients with psychiatric disorders (28–30), compared to healthy participants (31). At the same time, a systematic review has noted the susceptibility of perinatal women with psychological difficulties to avoidant behavioral patterns for stress coping (32, 33). Given that pregnant women are psychologically vulnerable under the pandemic, it is necessary to evaluate the roles of stress and behavioral responses manifested by their coping skills and their effects on perinatal mental health.

Since COVID-19 has a negative impact on pregnant women's perinatal mental health, the psychological demands of perinatal women need to be acknowledged. Based on our previous nationwide population-based cross-sectional survey, this study aims to investigate the possible role of different psychiatric diseases during the outbreak of COVID-19 and the influences on the behavioral changes and decision-making of prenatal examinations.

Therefore, a structural equation modeling (SEM) was defined to grasp the prediction of perinatal depression and PTSD during the COVID-19 outbreak. Based on the preceding literature review and objectives above, our primary hypotheses were as follows: (1) behavioral responses resulting from the pandemic are related to its psychological response, as well as probable PND and suspected PTSD; (2) behavioral responses and psychological responses resulting from the pandemic are interrelated to probable PND and suspected PTSD.

## Methods

### Study Design

A nationwide population-based cross-sectional study was conducted among pregnant women in China during the COVID-19 pandemic. Participants were invited to complete a web-based questionnaire. Participants were initially recruited from local hospitals across the nation via a hospital-based online education scheme for Chinese women during antenatal and postnatal periods. This online scheme allowed local hospitals to assess participating pregnant women's health conditions and provide primary care to their clients (*via* the Internet) during their pregnancy. Informed consent from each participant was obtained initially, prior to filling out the questionnaire. The data analyzed for this study was collected from 1908–2020, between February 13–16, from women in their second- or third-trimester of pregnancy.

### Research Protocol

The study protocol was reviewed and approved by the Institutional Review Board of the University of Hong Kong/Hospital Authority Hong Kong West Cluster (reference no: UW 20-252). All procedures were conducted in accordance with the Declaration of Helsinki.

### Measurements

Participants were given the following questionnaire in Chinese in a structured online-based questionnaire format:

#### Demographic Data

Participants' age, marital status, educational level, occupation, primiparity, presence of complications during pregnancy, and history of depression were collected.

#### Depression in Pregnancy

Depression in pregnancy or PND was assessed using the 10-item Chinese version of the Edinburgh Postnatal Depression Scale (EPDS), which was designed to assess perinatal depression to identify pregnant women at risk of PND (34, 35). This screening tool consists of 10 items relating to enjoyment and happiness, feelings of blame, anxiety and fear, sleeping problems, sadness, crying, and thoughts of self-harm. Each item is rated on a four-point scale from 0 to 3, and all items are added to form an overall score ranging from 0 to 30. Reverse scoring also applied in EPDS. Respondents are required to answer each question based on their feelings in the past 7 days. Participants scoring 13 or above are at considerable risk for depression, where probable or moderate PND is classified accordingly if the diagnosis is confirmed (36), and it is suggested to seek medical attention. Participants who scored between 10–12, indicate the experience of depressive symptoms and are classified as possible PND, which would be termed as mild PND if the diagnosis is confirmed (37). The internal consistency of the questionnaire was satisfactory (Cronbach α coefficient = 0.8480, Kaiser-Meyer-Olkin (KMO) measures of sampling adequacy = 0.881).

#### Post-traumatic Stress

Post-traumatic stress was measured using the PTSD Checklist-Specific version (PCL-S) in Chinese (38). The PCL asks respondents to indicate their level of irritation (five-point format) in response to specific stressful life experiences in the past month (39). For this study, the stressful experience was standardized as “the COVID-19 outbreak since late December 2019.” We used the abbreviated six-item version in this study (40). The six-items were derived from the original 17-item version with two items from each of three clusters (i.e., re-experiencing, avoidance, and hyperarousal) and have been validated for psychometry properties (*r* = 0.96–0.97 with the original total score). When using ≥ 14 as the cut-off, the checklist showed a sensitivity of 0.8–0.95 and specificity of 0.69–0.76 based on diagnostic interviews. This study adopted ≥ 14 as the cut-off point to classify participants into low or high PTSD risk (40). Due to the ongoing outbreak during data collection, those at higher risk (≥14) were demoted as suspected PTSD. The internal consistency of the questionnaire was good (Cronbach α-coefficient = 0.8253, KMO measures of sampling adequacy = 0.819).

#### COVID-Related Psychological and Behavioral Responses

We also evaluated multiple psychological and behavioral responses to COVID-19, including anxiety about the infection of members within one's social network (five questions), and specific psychological responses (anxiety when staying at home, lack of security, and loss of freedom; three questions). The internal consistency of the questionnaire was good (Cronbach α-coefficient = 0.8957, KMO measures of sampling adequacy = 0.868).

The behavioral response included items about precautionary behaviors (handwashing, use of facemasks, wearing gloves, and housebound behaviors; four questions) and psycho-behavioral responses, particularly related to pregnancy (anxiousness about miscarriage and premature birth, fear of antenatal check-up and consultation, and cancellation and postponement of antenatal visit; six questions). The internal consistency of the questionnaire was good (Cronbach α-coefficient = 0.4379, KMO measures of sampling adequacy = 0.691).

### Statistical Analyses

First, a descriptive statistical analysis of the main variables of interest was conducted, using central tendency (mean, median) and dispersion (standard deviation, interquartile interval) measures. In addition, preliminary regression analyses were performed to examine whether scores of scales differed by participants' demographics, and zero-order pair-wise correlations among them were reported. Finally, SEM, a type of multivariate analysis, was applied to confirm a theoretically built model that includes domains of post-traumatic stress experience, depression in pregnancy, COVID-19-related psychological and behavioral responses, as well as quality of life (QoL) domains.

In the first step, the model was designed and fitted with a well-defined research question. Next, the estimation and the significant levels for each parameter were obtained. Third, the assumed structure of the data was compared, and the goodness of fit measures for the model built were evaluated using multiple indices, including the chi-square statistic, the Comparative Fit Index (CFI), Tucker-Lewis Index (TLI), the χ^2^/d*f* , and the Root Mean Square Error of Approximation (RMSEA) (41). CFI ranged between 0 and 1, with values > 0.90 suggesting a good fit; RMSEA is a measure of the discrepancy between the model and the data per degree of freedom, with values <0.5 indicating close fit and values between 0.5 and 0.8 suggesting acceptable fit (42); and χ^2^/d*f* is a measure relating to the chi-square and degree of freedom, with the values less than three suggesting a good fit. If indicated, correlations between error terms were added and parameters were constrained to improve the model.

All analyses were conducted in R version 3.6.2. Statistical significance was set at *p* < 0.05.

## Results

### Demographic Characteristics

Table 1 summarizes the demographic characteristics of postpartum women completing the survey. Of the mothers completing the questionnaires (*n* = 1,908), 1,099 met the criteria for a positive screening for perinatal depression, and 287 met the criteria for a positive screening for PTSD, where 264 women exceed the cut-off points for both (Table 1). Moreover, the subsequent psychological and behavioral responses to the COVID-19 outbreak were designated as stress level and behavioral adjustment in the study and reported in Tables 2, 3, respectively. Married women were more likely to have PTSD experience and adopted preventive behaviors (13.27 ± 3.34, *p* = 0.011), yet single mothers had higher scores of EPDS (13.20 ± 5.78, *p* = 0.055). Pregnant women with full-time or part-time jobs tended to have the lowest scores of EPDS (10.07 ± 5.11, *P* < 0.001) and stress level (23.85 ± 7.96, *p* = 0.004), yet they were more likely to change their behavior in accordance with the outbreak of COVID-19 (13.35 ± 3.42, *p* = 0.025).

**Table 1:** Descriptive statistics of the demographic characteristics.

	**Demographic results (*N* = 1,908)**	
Age, mean (SD)		28.88 (4.75)
Whether in Hubei province, *n* (%)	Yes	95 (5.0%)
	No	1,813 (95.0%)
Education, n (%)	Primary school/ Junior Secondary school	397 (20.8%)
	Senior/ technical secondary school	474 (24.8%)
	College/ University	964 (50.5%)
	Graduate	73 (3.8%)
Marital state, *n* (%)	Single	15 (0.8%)
	Married/ cohabitation	1,893 (99.2%)
Gestation, median (IQR)		2 (1, 2)
Gravidity, median (IQR)		0 (0, 1)
Work, *n* (%)	Students/ not employed	76 (4.0%)
	Full-time/ part-time	930 (48.7%)
	Housewife	902 (47.3%)
Scores of PCL-6, mean (SD)		12.83 (4.17)
Scores of EPDS, mean (SD)		10.62 (5.21)
Scores of Behavioral response, mean (SD)		13.25 (3.36)
Scores of Psychological response level, mean (SD)		24.45(7.82)
EQ-VAS, mean (SD)		86.90 (14.22)
Probable PND, *n* (%)		1,099 (57.6%)
Suspected PTSD, *n* (%)		287 (15.0%)
Any Family infected, *n* (%)	Yes	2 (0.1%)
	No	1,906 (99.9%)
Any Friend infected, *n* (%)	Yes	11 (0.6%)
	No	1,897 (99.4%)
Any Neighbor infected, *n* (%)	Yes	40 (2.1%)
	No	1,868 (97.9%)
Ever Depression, *n* (%)	Yes	159 (8.3%)
	No	1,749 (91.7%)

**Table 2:** Demographic factors associated with the scores of scales.

***Educational level***	**Primary school/ Junior Secondary school (*N* = 397)**	**Senior/ technical secondary school (*N* = 474)**	**College/ University (*N* = 964)**	**Graduate (*N* = 73)**	***p*-value**
Scores of PTSD, mean (SD)	12.89 (4.47)	12.86 (3.95)	12.83 (4.19)	12.18 (3.69)	0.6
Scores of EPDS, mean (SD)	11.39 (5.43)	10.97 (4.94)	10.30 (5.24)	8.53 (4.45)	<0.001
Suspected PTSD, *n* (%)	61 (15.4%)	73 (15.4%)	146 (15.1%)	7 (9.6%)	0.62
Probable PND, *n* (%)	255 (64.2%)	288 (60.8%)	528 (54.8%)	28 (38.4%)	<0.001
Behavioral responses, mean (SD)	13.21 (3.45)	13.17 (3.29)	13.30 (3.37)	13.29 (3.20)	0.89
Psychological responses, mean (SD)	25.50 (7.52)	25.22 (6.91)	23.82 (8.25)	22.04 (7.90)	<0.001
***Occupational states***	**Students/ not employed (*****N*** **=** **76)**	**Full-time/ part-time (*****N*** **=** **930)**	**Housewife (*****N*** **=** **902)**		***p*** **-value**
Scores of PTSD, mean (SD)	12.64 (4.44)	12.72 (4.11)	12.95 (4.22)		0.49
Scores of EPDS, mean (SD)	11.96 (5.95)	10.07 (5.11)	11.09 (5.18)		<0.001
Suspected PTSD, *n* (%)	9 (11.8%)	127 (13.7%)	151 (16.7%)		0.13
Probable PND, *n* (%)	50 (65.8%)	491 (52.8%)	558 (61.9%)		<0.001
Behavioral responses, mean (SD)	12.26 (3.92)	13.35 (3.42)	13.23 (3.23)		0.025
Psychological responses, mean (SD)	24.76 (8.63)	23.85 (7.96)	25.04 (7.55)		0.004
***Marital status***	**Single (*****N*** **=** **15)**	**Married (*****N*** **=** **1,893)**			***p*** **-value**
Scores of PTSD, mean (SD)	11.33 (4.75)	12.84 (4.17)			0.16
Scores of EPDS, mean (SD)	13.20 (5.78)	10.60 (5.20)			0.055
Suspected PTSD, n (%)	2 (13.3%)	285 (15.1%)			0.85
Probable PND, *n* (%)	11 (73.3%)	1,088 (57.5%)			0.22
Behavioral responses, mean (SD)	11.07 (4.67)	13.27 (3.34)			0.011
Psychological responses, mean (SD)	24.47 (7.93)	24.45 (7.82)			0.99

**Table 3:** The pair-wise Pearson correlation of the measured variables.

**Variables**	**(1)**	**(2)**	**(3)**	**(4)**	**(5)**	**(6)**	**(7)**	**(8)**	**(9)**	**(10)**	**(11)**	**(12)**	**(13)**	**(14)**	**(15)**	**(16)**
(1) Scores of PCL−6	1.000															
(2) Scores of EPDS	0.626^***^	1.000														
(3) Scores of Psychological response	0.520^***^	0.504^***^	1.000													
(4) Scores of Behavioral responses	0.211^***^	0.211^***^	0.325^***^	1.000												
(5) Age	0.014	−0.081^***^	−0.068^**^	0.132^***^	1.000											
(6) Education	−0.016	−0.109^***^	−0.108^***^	0.014	0.121^***^	1.000										
(7) Marital state	−0.032	0.044	0.000	−0.058	−0.035	−0.060^**^	1.000									
(8) Number of gestations	0.032	0.045	0.000	0.010	0.262^***^	−0.232^***^	0.004	1.000								
(9) Number of gravidities	0.055	0.068^**^	0.021	0.072^**^	0.231^***^	−0.275^***^	0.024	0.341^***^	1.000							
(10) Any pregnancy complications	0.079^*^	0.085^***^	0.044	0.028	0.063^**^	−0.014	0.006	0.027	0.017	1.000						
(11) Any family infected	−0.053	−0.004	0.004	0.012	0.100^***^	−0.014	0.181^***^	0.034	0.088^***^	0.040	1.000					
(12) Any friend infected	−0.008	0.001	−0.008	−0.012	0.015	−0.025	0.072^**^	0.008	0.048	−0.005	0.425^***^	1.000				
(13) Any neighbor infected	0.026	0.022	0.024	0.019	0.020	0.043	0.028	−0.020	0.016	0.006	0.108^***^	0.037	1.000			
(14) Any depression history	0.131^***^	0.204^**^	0.054	−0.012	−0.057	−0.025	0.016	0.019	0.036	0.030	0.049	0.002	−0.004	1.000		
(15) QoL (EQVAS)	−0.363^***^	−0.461^***^	−0.218^***^	−0.024	0.055	0.007	−0.017	0.000	0.009	−0.069^**^	−0.019	−0.036	0.001	−0.202^***^	1.000	
(16) Whether in Hubei	0.066^**^	0.019	0.028	0.007	0.005	−0.021	0.034	−0.004	−0.023	0.010	0.067^**^	0.078^***^	0.236^***^	−0.017	0.013	1.000

*** *p<0.01*,

** *p<0.05*,

* *p<0.1*.

The influence of demographic factors on the scores of scales was reported in Table 2, where the educational level, occupational status, and marital status were included. We found that women with lower educational levels tended to have higher scores of PCL-6, and EPDS scales as well as stress level and behavioral adjustment, and more were regarded as suspected PTSD and probable PND. Nevertheless, compared to the level-dependent pattern between education level and scores of scales, the occupational states were paradoxically associated with the scores. Pregnant women with full-time or part-time jobs tended to have the lowest scores of EPDS (10.0 ± 5.11, *p* < 0.001) and stress level (23.85 ± 7.96, *p* = 0.004), yet they were more likely to change their behavior in accordance with the outbreak of COVID-19 (13.35 ± 3.42, *p* = 0.025). Housewives had a higher level of PTSD experience but a medium level of EPDS scores. Still, married women were more likely to have PTSD experience and adopted preventive behaviors (13.27 ± 3.34, *p* = 0.011), yet the single mother had higher scores of EPDS (13.20 ± 5.78, *p* = 0.055).

### Preliminary Analyses and Pearson Correlation Coefficients

Generalized linear modeling (GLM) multivariate test results showed that the four scores of the scales have distinct patterns of difference by marital status, employment status, education level, obstetrics history (gestation, gravidity, and any pregnancy complication history), any history of COVID-19 exposure (any history of friend, family, or neighbors infected), and whether in Wuhan (Supplementary Tables S1–4). First, age (Coef = −0.09, *t*-value = −3.22, *p* = 0.001), number of gravidities (Coef = 0.347, *t*-value = 3.94, *p* = 0.022), history of any pregnancy complications (Coef = 1.477, *t*-value = 3.94, *p* < 0.001), and education were significant predictors for the scores of EPDS (Supplementary Table S1), where a higher level of education associated with lower scores of EPDS. In addition, being in Hubei (Coef = 1.251, *t*-value = 2.77, *p* = 0.006), number of gravidity (Coef = 0.275, *t*-value = 2.25, *p* = 0.024), and history of any pregnancy complications (Coef = 1.08, *t*-value = 3.57, *p* < 0.001) served as significant predictors for the scores of PCL-6 (Supplementary Table S2). Moreover, the scores of the COVID-19-related psychological responses were significantly related to age (Coef = −0.088, *t*-value = −2.09, *p* = 0.037), history of any pregnancy complications (Coef = 1.229, *t*-value = 2.17, *p* = 0.118), and education level (Supplementary Table S3). Finally, age (Coef = 0.086, *t*-value = 4.79, *p* < 0.001), marital status (Coef = −1.951, *t*-value = −2.19, *p* = 0028), and number of gravidity (Coef = 0.229, *t*-value = 2.33, *p* = 0.02) served as significant predictors for the scores of COVID-19-related behavioral responses (Supplementary Table S4).

The correlation for all indicators is shown in Table 3. The scores of the four scales were significantly correlated, and the QoL, as measured by EQVAS, was also significantly correlated with the scores of PCL-6, EPDS, and COVID-19-related psychological responses. Specifically, past-history of depression was also found to significantly correlated with the scores of PCL-6 and scores of EPDS. Age was also significantly correlated with the scores of EPDS and scores of COVID-19-related psychological and behavioral responses. Moreover, being in Hubei Province, the epicenter of the COVID-19 pandemic in China, was found to be significantly correlated with the scores of PCL-6, and the history of any pregnancy complications was also found to be significantly correlated with the scores of PCL-6 and EPDS.

### Structural Equation Modeling

Structural equation modeling was conducted to evaluate the proposed structural model. First, to evaluate the intercorrelation between pregnant women's psychological and behavioral responses during the COVID-19 pandemic. The QoL score was hypothesized to have direct and indirect effects on PND and PTSD, as well as psychological and behavioral responses. Second, the state of PND was hypothesized to have direct effects on the post-traumatic stress experience, psychological responses, and behavioral responses. Moreover, behavioral responses were hypothesized to be influenced by PND and directly influenced psychological responses.

**Figure 1:**
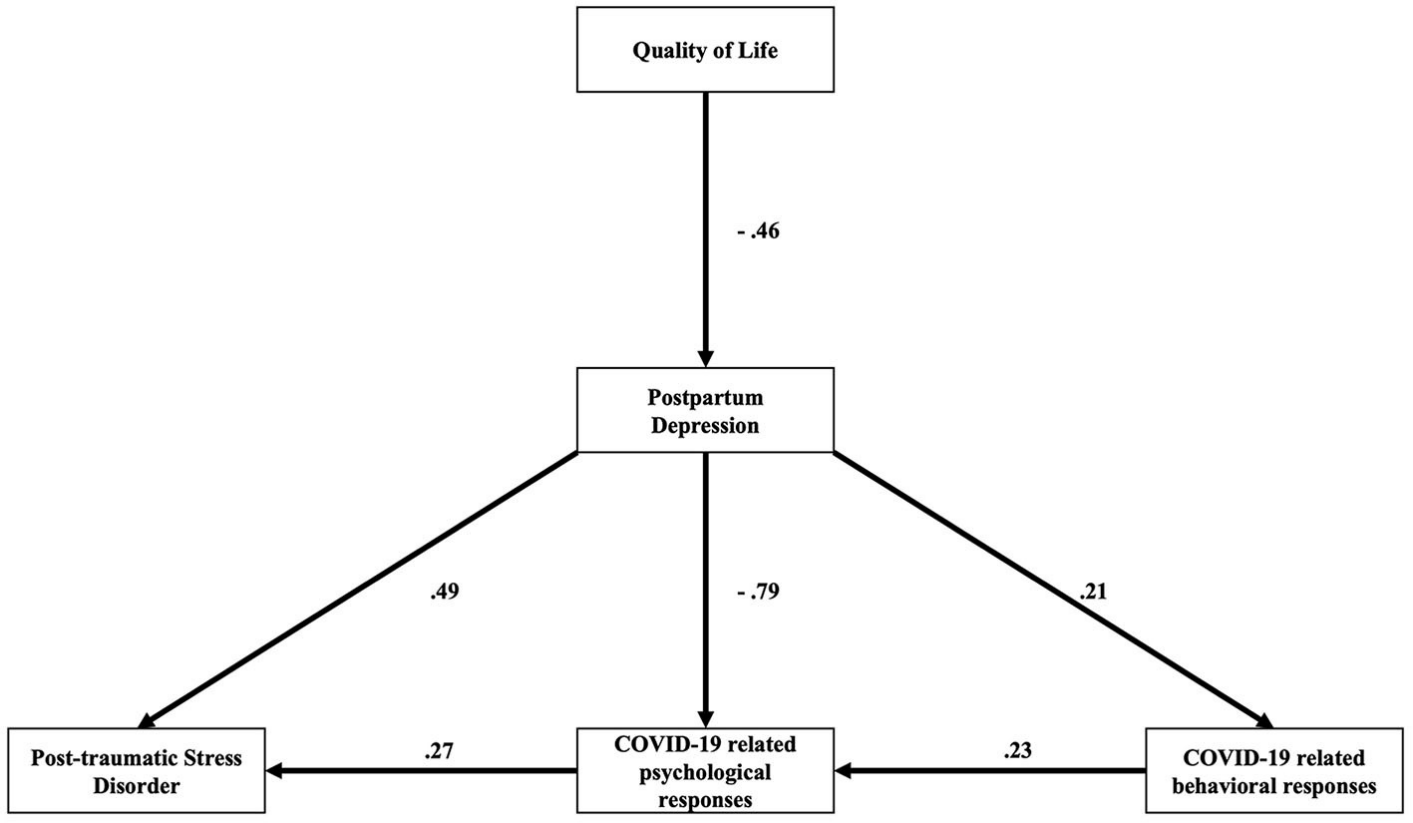
The hypothesized model relating the scores of PCL-6, EPDS, and COVID-9-related psychological and behavioral. All path loadings were significant (*p* < 0.05).

Figure 1 shows significant pathways of the final model. The structural model fit the data (χ^2^ = 43.260, *p* < 0.001) and resulted in the following satisfactory fit indices (CFI = 0.984, TLI = 0.959, RMSEA = 0.072, and χ^2^/d*f* = 10.815). All path loadings were significant (*p* < 0.05), and the results of the SEM are reported in Table 4.

**Table 4:** Relationship between independent and dependent variables.

			**Estimate**	**Standard errors**	***z*-value**	***p*-value**	**95% Confidence interval**	
Scores of PCL−6	–>	Scores of Psychological responses	0.147	0.011	13.959	0	0.126	0.167
Scores of PCL−6	–>	Scores of EPDS	0.391	0.016	24.788	0	0.360	0.421
Scores of Psychological responses	–>	Scores of Behavioral responses	0.533	0.045	11.726	0	0.444	0.622
Scores of Psychological responses	–>	Scores of EPDS	0.682	0.029	23.282	0	0.625	0.740
Scores of Behavioral responses	–>	Scores of EPDS	0.136	0.014	9.452	0	0.108	0.165
Scores of EPDS	–>	Scores of EQVAS	−0.169	0.007	−22.712	0	−0.184	−0.154
Scores of PCL−6	~~	Scores of PCL−6	9.597	0.311	30.887	0	8.988	10.206
Scores of Psychological responses	~~	Scores of Psychological responses	42.503	1.376	30.887	0	39.806	45.200
Scores of Behavioral responses	~~	Scores of Behavioral responses	10.769	0.349	30.887	0	10.086	11.453
Scores of EPDS	~~	Scores of EPDS	21.367	0.692	30.887	0	20.011	22.722

## Discussion

To our knowledge, this is the first SEM study based on real-world data from a nationwide study to examine the relationship between various psychological factors and behavioral changes in pregnant women during the COVID-19 outbreak. The survey was conducted during the peak (between February 13 and 16, 2020) of the COVID-19 outbreak in China. Our study demonstrates the relationship between post-traumatic experience, depression in pregnancy, and the interrelationship between behavioral and psychological responses in pregnant women.

The COVID-19 outbreak had profound psychological impacts on pregnant women. A substantial portion of pregnant women have demonstrated to have suspected PTSD, probable PPD symptoms, high-stress levels, and subsequent behavioral adjustment. As much as 24.8% of pregnant women considered themselves likely to be infected by SARS-CoV-2 (15). Incidents illustrating a biased risk assessment over COVID-19 could be seen in avoidance behavior, such as canceling and postponing antenatal check-up appointments. About 71.2% of pregnant women reported having canceled or postponed antenatal check-up appointments during the outbreak. These findings are consistent with a survey on psychological response, behavior, and attitudes of pregnant women to the SARS outbreak by Ng et al. (5) and Lee et al. (9). Although a recent investigation on the public's psychological responses during the initial stage of COVID-19 in China reported that most respondents believed the risk of contracting the infection during the outbreak was low (43), pregnant women we surveyed were highly stressed, possibly owing to the pressure of decision-making on the undergoing of antenatal examinations, risking viral exposure in hospital settings. Psychological effects brought about by public health measures may also contribute to stress production (44).

Secondly, demographic factors have been found to influence women's symptoms, as shown in the scores of scales in Table 4, where educational level, occupational status, and marital status were the most notable. Women with lower educational levels tended to have higher scores of PCL-6 and EPDS scales, as well as psychological and behavioral responses during the COVID-19 pandemic, where more than 15% and 60% of those with only primary school and junior secondary school education have been regarded as suspected PTSD and probable PND, respectively. Such findings correlate with the study by Hanna-Leena et al. (45), where they reported that the most important factors contributing to perinatal anxiety included employment and elective cesarean section, while educational levels played a minor role (45). Therefore, improving antenatal emotional well-being may have positive social and maternal care for optimal childbirth experiences, especially during the COVID-19 outbreak. A clinical trial by Jocelyn et al. (46) reported that pregnant women with psycho-education by trained midwives had an effective reduction of fear that may be associated with the delivery and elevated childbirth confidence (46). Hence, greater social support might also help to buffer depression and anxiety-related symptoms in pregnant women (47).

Psychologists have published a wide range of theories in attempt to explain the underlying mechanism of infectious outbreak-related anxiety and fear. Anxiety and fear are implicitly results of biased information processing (48), but also in explicit biases in the evaluation of risk and information (49, 50). When risks regarding an event are overestimated in its likelihood of occurrence or its severity, a maladaptive fear process can generate, leading to the irrationality of one's anticipatory response (51, 52).

As none of the 1908 participants had contracted the infection, the pregnant women may, in comparison to the public, have overestimated the risk of COVID-19. Nevertheless, their high-stress level should not be neglected. Disease outbreaks related to anxiety and panic are attributable to two factors: stigma on the infectious virus and fear of the unknown (50). The effect of the stigma attached to the virus is widely documented in population survey studies on outbreaks, including SARs, Ebola, and Zika. Fear of the unknown might be provoked by unforeseeable events (such as death or disabilities), the unknown nature of the virus, and economic paralysis (53, 54).

Moreover, healthcare authorities need to provide psychological support for those in need. Online and smartphone-based psychological interventions, including hotlines to provide crisis support should be considered. The content of these interventions needs to be modified to suit the needs of the pregnant women presenting with mental health difficulties during the pandemic. Physical symptoms, as a result of psychological distress should also be addressed. Symptoms such as headaches, fatigue, dizziness, high blood pressure, and breathing difficulties are often presented in people with mental illnesses, such as anxiety, depression, and PTSD. It is evident that those with poor health status and a history of chronic illness are more likely to experience psychological impacts as well as stress, anxiety, and depression amidst an outbreak (55). Health professionals should be aware of the symptoms above and provide early adequate psychological support and intervention. Moreover, healthcare authorities should be alert to possible long-term psychiatric sequelae, especially PTSD and PPD.

There are several limitations to this study. The questionnaire was self-administered and hence, reporting bias is possible. The study measured the stress level and behavioral adjustment using a newly developed questionnaire. Though we have tested the validity and reliability of the questionnaire, there still may be unfound bias and limited utility in reflecting the real conditions. The comprehension of the questionnaire by the respondents was not evaluated. It was not possible to evaluate psychometric performance of the questionnaire because the questionnaire is simply a collection of questions on how pregnant women reacted to the outbreak. However, the internal consistency of the questionnaire has been assessed and resulted in promising reliability. The psychological responses of pregnant women toward the infection were collected prior to its declaration as a pandemic. Thus, only the initial and intermediary responses were examined in the present study. Moreover, though information on education level and culture were collected, we failed to investigate the socioeconomic status or collect data on income. All the respondents in the study were pregnant Chinese women involved in an online teaching school, which may limit the homogeneity of the respondents. A longitudinal follow-up investigation is pending to examine the long-term psychological effects of this outbreak.

## Conclusion

To date, the psychological stress of pregnant women under the COVID-19 pandemic have been underestimated. Our results contributed to elucidating the relationships between the COVID-19-related distress and the mental health of women in the perinatal period. Perinatal depression is associated with PTSD-like anxiety and may lead to COVID-19-related behavioral and psychological responses in pregnant women during the COVID-19 pandemic. Mental health surveillance in pregnant women is necessary. Psychoeducation, as well as other psychological interventions, may be needed to equip pregnant women and their family members with problem-solving and communication skills to alleviate COVID-19-related distress.

## Significant Outcomes

Out of the 1,908 mothers who completed the questionnaires, 1,099 met the criteria for perinatal depression, and 287 were positively screened for PTSD where 264 women exceed the cut-off points for both.

Being in Hubei Province, the epicenter of the COVID-19 pandemic in China, was found to be significantly correlated with the scores of PCL-6, and the history of any pregnancy complications was also found to be significantly correlated with the scores of PCL-6 and EPDS.

Moreover, the scores of the COVID-19-related psychological responses were significantly related to age, history of any pregnancy complications, and education level.

## Take-Home Messages

With the COVID-19 pandemic ongoing, and normalized regulatory stage enforced, the COVID-19 outbreak represented an additional factor that increased the burden of perinatal depression, and psychoeducation, as well as other psychological interventions, may be needed to improve their mental health.

## Limitations

The questionnaire was self-administered, and hence, reporting bias is possible.

The study measured stress levels and behavioral adjustment using a newly developed questionnaire. Though we have tested the validity and reliability of the questionnaire, unfound bias and limited utility in reflecting actual conditions may remain.

Psychological responses of pregnant women toward the infection were collected prior to its declaration as a pandemic, and a follow-up investigation was not possible.

## Data Availability Statement

The raw data supporting the conclusions of this article will be made available by the authors, without undue reservation.

## Ethics Statement

The studies involving human participants were reviewed and approved by UW 20-252. The patients/participants provided their written informed consent to participate in this study.

## Author Contributions

ZH and JC conceptualized the idea, established the questionnaire, and analyzed the data, as well as drafted the manuscript. YL, BA, TW, CZ, and W-KM drafted and revised the manuscript, while helping to perform the statistical analysis and produce the graphics. All authors contributed to the article and approved the submitted version.

## Funding

This work was funded by the City University of Hong Kong New Research Initiatives/Infrastructure Support from Central (APRC; grant number 9610589).

## Conflict of Interest

The authors declare that the research was conducted in the absence of any commercial or financial relationships that could be construed as a potential conflict of interest.

## Publisher's Note

All claims expressed in this article are solely those of the authors and do not necessarily represent those of their affiliated organizations, or those of the publisher, the editors and the reviewers. Any product that may be evaluated in this article, or claim that may be made by its manufacturer, is not guaranteed or endorsed by the publisher.

## Abbreviations

SARS: Severe Acute Respiratory Syndrome
COVID-19: Coronavirus disease 2019
PTSD: post-traumatic stress disorder
PND: prenatal depression
SEM: structural equation modeling
EPDS: Edinburgh Postnatal Depression Scale
PCL-S: PTSD Checklist-Specific version
QoL: quality of life
CFI: Comparative Fit Index
TLI: Tucker-Lewis Index
RMSEA: Root Mean Square Error of Approximation.
