# Supplementary material for: The Construct Structures of Psychological and Behavioral Responses to COVID-19 Pandemic in Pregnant Women
Source: Front Psychiatry. 2022 Jul 12;13:796567. doi: 10.3389/fpsyt.2022.796567 (PMC9323087; doi:10.3389/fpsyt.2022.796567)
Supplement: Supplementary file 1 [file Data_Sheet_1.docx]

**Supplementary Tables:**

**Supplementary Table 1.** **Generalized linear models of Scores of EPDS and demographic factors.**

| **Scores of EPDS** | **Coef.** | | **St.Err.** | **t-value** | | **p-value** | **[95% Conf** | | **Interval]** | | **Sig** |  |
| --- | --- | --- | --- | --- | --- | --- | --- | --- | --- | --- | --- | --- |
| Whether in Hubei | .237 | | .561 | 0.42 | | .672 | -.861 | | 1.336 | |  |  |
| Age | -.09 | | .028 | -3.22 | | .001 | -.144 | | -.035 | | *** |  |
| Marital state | 1.902 | | 1.374 | 1.38 | | .166 | -.792 | | 4.596 | |  |  |
| Number of gestation | .103 | | .088 | 1.17 | | .242 | -.069 | | .274 | |  |  |
| Number of gravidity | .347 | | .152 | 2.29 | | .022 | .05 | | .644 | | ** |  |
| Any pregnant complications | 1.477 | | .375 | 3.94 | | 0 | .741 | | 2.212 | | *** |  |
| Any family infected | -2.833 | | 4.138 | -0.68 | | .494 | -10.943 | | 5.277 | |  |  |
| Any friend infected | .098 | | 1.723 | 0.06 | | .955 | -3.279 | | 3.475 | |  |  |
| Any neighbor infected | .84 | | .852 | 0.99 | | .325 | -.831 | | 2.51 | |  |  |
| Work_unemployed | .657 | | .63 | 1.04 | | .297 | -.578 | | 1.892 | |  |  |
| Work_working | -.489 | | .278 | -1.76 | | .079 | -1.034 | | .056 | | * |  |
| Education_ Primary school/ Junior high school | 1.738 | | .704 | 2.47 | | .014 | .357 | | 3.119 | | ** |  |
| Education_Senior high school | 1.539 | | .679 | 2.27 | | .023 | .208 | | 2.87 | | ** |  |
| Education_Undergraduate | 1.29 | | .631 | 2.04 | | .041 | .053 | | 2.527 | | ** |  |
| Constant | 11.41 | | 1.036 | 11.01 | | 0 | 9.38 | | 13.441 | | *** |  |
|  | | | | | | | | | | | | |
| Mean dependent var | | 10.627 | | | SD dependent var | | | 5.214 | |  |  |  |
| Number of obs | | 1905 | | | Chi-square | | | 69.018 | |  |  |  |
| Prob > chi2 | | 0.000 | | | Akaike crit. (AIC) | | | 11658.523 | |  |  |  |
| **** p<.01, ** p<.05, * p<.1* | | | | | | | | | | | | |
|  | | | | | | | | | | | | |

**Supplementary Table 2.** **Generalized linear models of Scores of PCL-6 and demographic factors.**

| **Scores of PCL-6** | **Coef.** | | **St.Err.** | **t-value** | | **p-value** | **[95% Conf** | | **Interval]** | | **Sig** |
| --- | --- | --- | --- | --- | --- | --- | --- | --- | --- | --- | --- |
| Whether in Hubei | 1.251 | | .452 | 2.77 | | .006 | .365 | | 2.138 | | *** |
| Age | .002 | | .022 | 0.08 | | .937 | -.042 | | .046 | |  |
| Marital state | -1.18 | | 1.109 | -1.06 | | .287 | -3.353 | | .993 | |  |
| Number of gestation | .039 | | .071 | 0.55 | | .584 | -.1 | | .177 | |  |
| Number of gravidity | .275 | | .122 | 2.25 | | .024 | .036 | | .515 | | ** |
| Any pregnant complications | 1.08 | | .303 | 3.57 | | 0 | .487 | | 1.673 | | *** |
| Any family infected | -9.005 | | 3.338 | -2.70 | | .007 | -15.548 | | -2.462 | | *** |
| Any friend infected | .82 | | 1.39 | 0.59 | | .555 | -1.905 | | 3.544 | |  |
| Any neighbor infected | .494 | | .688 | 0.72 | | .472 | -.854 | | 1.842 | |  |
| Work_unemployed | -.001 | | .508 | -0.00 | | .999 | -.997 | | .996 | |  |
| Work_working | -.163 | | .224 | -0.73 | | .467 | -.603 | | .277 | |  |
| Education_ Primary school/ Junior high school | .252 | | .568 | 0.44 | | .657 | -.862 | | 1.366 | |  |
| Education_Senior high school | .378 | | .548 | 0.69 | | .491 | -.696 | | 1.451 | |  |
| Education_Undergraduate | .454 | | .509 | 0.89 | | .373 | -.545 | | 1.452 | |  |
| Constant | 12.052 | | .836 | 14.42 | | 0 | 10.414 | | 13.69 | | *** |
|  | | | | | | | | | | | |
| Mean dependent var | | 12.828 | | | SD dependent var | | | 4.174 | |  |  |
| Number of obs | | 1905 | | | Chi-square | | | 39.192 | |  |  |
| Prob > chi2 | | 0.000 | | | Akaike crit. (AIC) | | | 10840.501 | |  |  |
| **** p<.01, ** p<.05, * p<.1* | | | | | | | | | | | |
|  | | | | | | | | | | | |

**Supplementary Table 3. Generalized linear models of Scores of COVID-19-related psychological responses and demographic factors.**

| **Psychological responses** | **Coef.** | | **St.Err.** | **t-value** | | **p-value** | **[95% Conf** | | **Interval]** | | **Sig** |
| --- | --- | --- | --- | --- | --- | --- | --- | --- | --- | --- | --- |
| Whether in Hubei | .652 | | .846 | 0.77 | | .441 | -1.007 | | 2.311 | |  |
| Age | -.088 | | .042 | -2.09 | | .037 | -.17 | | -.005 | | ** |
| Marital state | -.912 | | 2.075 | -0.44 | | .66 | -4.979 | | 3.155 | |  |
| Number of gestation | -.069 | | .132 | -0.52 | | .604 | -.328 | | .191 | |  |
| Number of gravidity | .096 | | .229 | 0.42 | | .675 | -.353 | | .544 | |  |
| Any pregnant complications | 1.229 | | .567 | 2.17 | | .03 | .118 | | 2.339 | | ** |
| Any family infected | 2.807 | | 6.248 | 0.45 | | .653 | -9.439 | | 15.054 | |  |
| Any friend infected | -1.645 | | 2.602 | -0.63 | | .527 | -6.744 | | 3.454 | |  |
| Any neighbor infected | 1.353 | | 1.287 | 1.05 | | .293 | -1.17 | | 3.875 | |  |
| Work_unemployed | -.272 | | .951 | -0.29 | | .775 | -2.137 | | 1.592 | |  |
| Work_working | -.273 | | .42 | -0.65 | | .515 | -1.096 | | .55 | |  |
| Education_Primary school/ Junior high school | 2.987 | | 1.064 | 2.81 | | .005 | .903 | | 5.072 | | *** |
| Education_Senior high school | 2.7 | | 1.025 | 2.63 | | .008 | .691 | | 4.71 | | *** |
| Education_Undergraduate | 1.452 | | .953 | 1.52 | | .128 | -.417 | | 3.32 | |  |
| Constant | 25 | | 1.564 | 15.98 | | 0 | 21.934 | | 28.066 | | *** |
|  | | | | | | | | | | | |
| Mean dependent var | | 24.459 | | | SD dependent var | | | 7.814 | |  |  |
| Number of obs | | 1905 | | | Chi-square | | | 39.645 | |  |  |
| Prob > chi2 | | 0.000 | | | Akaike crit. (AIC) | | | 13228.809 | |  |  |
| **** p<.01, ** p<.05, * p<.1* | | | | | | | | | | | |
|  | | | | | | | | | | | |

**Supplementary Table 4. Generalized linear models of Scores of COVID-19-related behavioral responses and demographic factors.**

| **Behavioral responses** | **Coef.** | | **St.Err.** | **t-value** | | **p-value** | **[95% Conf** | | **Interval]** | | **Sig** |
| --- | --- | --- | --- | --- | --- | --- | --- | --- | --- | --- | --- |
| Whether in Hubei | .066 | | .363 | 0.18 | | .856 | -.646 | | .777 | |  |
| Age | .086 | | .018 | 4.79 | | 0 | .051 | | .122 | | *** |
| Marital state | -1.951 | | .89 | -2.19 | | .028 | -3.695 | | -.207 | | ** |
| Number of gestation | -.091 | | .057 | -1.61 | | .107 | -.203 | | .02 | |  |
| Number of gravidity | .229 | | .098 | 2.33 | | .02 | .036 | | .421 | | ** |
| Any pregnant complications | .228 | | .243 | 0.94 | | .349 | -.248 | | .704 | |  |
| Any family infected | 1.35 | | 2.679 | 0.50 | | .614 | -3.9 | | 6.601 | |  |
| Any friend infected | -.804 | | 1.115 | -0.72 | | .471 | -2.99 | | 1.382 | |  |
| Any neighbor infected | .333 | | .552 | 0.60 | | .546 | -.749 | | 1.414 | |  |
| Work_unemployed | -.572 | | .408 | -1.40 | | .161 | -1.371 | | .228 | |  |
| Work_working | -.049 | | .18 | -0.27 | | .787 | -.401 | | .304 | |  |
| Education_ Primary school/ Junior high school | .093 | | .456 | 0.20 | | .839 | -.801 | | .987 | |  |
| Education_Senior high school | .17 | | .44 | 0.39 | | .699 | -.692 | | 1.032 | |  |
| Education_Undergraduate | .159 | | .409 | 0.39 | | .698 | -.642 | | .96 | |  |
| Constant | 10.676 | | .671 | 15.92 | | 0 | 9.362 | | 11.991 | | *** |
|  | | | | | | | | | | | |
| Mean dependent var | | 13.252 | | | SD dependent var | | | 3.360 | |  |  |
| Number of obs | | 1905 | | | Chi-square | | | 50.642 | |  |  |
| Prob > chi2 | | 0.000 | | | Akaike crit. (AIC) | | | 10002.115 | |  |  |
| **** p<.01, ** p<.05, * p<.1* | | | | | | | | | | | |
|  | | | | | | | | | | | |
